# Boson peak, heterogeneity and intermediate-range order in binary SiO_2_-Al_2_O_3_ glasses

**DOI:** 10.1038/s41598-018-23574-1

**Published:** 2018-03-29

**Authors:** Mariana F. Ando, Omar Benzine, Zhiwen Pan, Jean-Luc Garden, Katrin Wondraczek, Stephan Grimm, Kay Schuster, Lothar Wondraczek

**Affiliations:** 1 Otto Schott Institute of Materials Research, University of Jena, 07743 Jena, Germany; 2 Institut NÉEL, CNRS, 38042 Grenoble, France; 3 University Grenoble Alpes, Institut NÉEL, 38042 Grenoble, France; 4 Leibniz Institute of Photonic Technology, 07745 Jena, Germany; 5 Abbe Center of Photonics, University of Jena, 07745 Jena, Germany

## Abstract

In binary aluminosilicate liquids and glasses, heterogeneity on intermediate length scale is a crucial factor for optical fiber performance, determining the lower limit of optical attenuation and Rayleigh scattering, but also clustering and precipitation of optically active dopants, for example, in the fabrication of high-power laser gain media. Here, we consider the low-frequency vibrational modes of such materials for assessing structural heterogeneity on molecular scale. We determine the vibrational density of states VDoS g(ω) using low-temperature heat capacity data. From correlation with low-frequency Raman spectroscopy, we obtain the Raman coupling coefficient. Both experiments allow for the extraction of the average dynamic correlation length as a function of alumina content. We find that this value decreases from about 3.9 nm to 3.3 nm when mildly increasing the alumina content from zero (vitreous silica) to 7 mol%. At the same time, the average inter-particle distance increases slightly due to the presence of oxygen tricluster species. In accordance with Loewensteinian dynamics, this proves that mild alumina doping increases structural homogeneity on molecular scale.

## Introduction

At low temperature (<50 K), the heat capacity *C*_p_ of glasses and other non-crystalline materials exhibits a characteristic deviation from the Debye model^1^. The underlying excess of vibrational states is often taken as a universal signature of structural heterogeneity at intermediate length scale^2^, resulting from dynamic heterogeneity in the liquid state which leads to spatial gradients in fictive temperature^3,4^. Heterogeneity then manifests not only in the excess of heat capacity, but also in the terahertz frequency range (0.1–3 THz) which is detectable by low-frequency Raman spectroscopy^5^, or in the inelastic scattering of X-rays (IXS) and neutrons (INS)^6,7^. While the exact consequences of such intermediate-range structural features remain unclear, various links have been identified with macroscopic properties, *e*.*g*., optical attenuation^8^, mechanical behaviour^9^ or ion mobility^10^.

Here, we consider the binary system of SiO_2_-Al_2_O_3_, both as a fundamental model for a mixed tetrahedral network^11^ and for the wide relevance of aluminosilicates, ranging from geosciences to optical fiber and high-power laser gain media^12^. In the latter, alumina is one of the most significant dopants used in silica-based active fiber laser applications. This is primarily for improving the solubility of rare-earth ions and to prevent their clustering^13^. However, typical strategies for compositional design remain empirical due to the lack of understanding regarding the structural role of alumina on intermediate length scale. On the other hand, high dopant capacity allows for shorter fiber lengths and/or significantly higher power levels^14^. We now  explore the low-frequency modes of binary aluminosilicate glasses through analysis of low temperature heat capacity and low frequency Raman scattering so as to find quantitative scaling parameters and, subsequently, relations between chemical composition, intermediate-range order and the length-scale of elastic heterogeneity. This is to enable a design strategy for glasses with high dopant capacity, but also to provide new insight at the structural origin of the excess in the vibrational density of states and the Boson peak of aluminosilicate materials.

## Experimental

Binary (100-x) SiO_2_ – x Al_2_O_3_ glasses with 2.05 < × < 7 mol % were prepared by reactive powder sintering of nanoscale silica^15–17^. Basically, this method is a modification of the solution-doping process such as employed for the production of alumina- or rare-earth-doped silica for optical fiber preforms. In the present case, sample fabrication involved the preparation of a porous green body from Al-doped high-purity silica nanopowder with a BET surface area of ~60–76 m²/g. For this, an aqueous silica slurry was doped with appropriate amounts of aqueous AlCl_3_ solution (1 M; AlCl_3_*6H_2_O 99.9995% metals basis, Alpha Aesar). The slurry mixture was cast into molds, dried and isostatically pressed to yield rod-shaped preform bodies of about 20 mm in diameter and a length of a few centimeters. The subsequent sintering and vitrification process of the preforms was carried-out in silica jacketing tubes (Heraeus F300) by applying mild vacuum and temperatures of up to 2200 °C (obtained from passing an oxygen-hydrogen burner). The subsequent sintering and vitrification process was carried out at temperatures of up to 2200 °C. Chemical analysis of all such-derived samples was conducted through wavelength-dispersive electron probe microanalysis (WD-EMPA). Besides the primary constituents, impurities of chlorine and water were present in the samples with average concentrations in the doped zone of less than 0.15 mol% (SiCl_4_) and <10 ppm (OH, determined by infrared optical spectroscopy). In the following, samples are denoted according to their Al_2_O_3_ concentration, *x* = 0, 2.05, 3.20 and 7.00, (Table 1). Sample homogeneity was examined by visual inspection, optical microscopy and through transmission electron microscopy (TEM-EDS). It was confirmed that the samples do not contain any kind of crystals, pores or bubbles. Furthermore, on a length-scale of 5–3000 nm, they did not contain any visible fluctuations in chemical composition or density.

**Table 1:** Density *ρ*, longitudinal sound velocity *v*_*L*_, transversal sound velocity *v*_*T*_, Debye temperature *θ*_*D*_, Debye frequency ω_*D*_, and location of the Boson peak maximum in the plot of *C*_p_(*T*)/*T*^3^ over *T*, *T*_*max*_, bulk modulus *K*, *poisson ratio v and Young’s modulus E* of silica and binary aluminosilicate glasses investigated in this study.

Al_2_O_3_ (mol %)	ρ (g/cm^3^)	K (GPa)	E (GPa)	ν	v_L_(ms^−1^)	v_T_ (ms^−1^)	θ_D_ (K)	ω_D_ (THz)	T_max_ (K)
0.00	2.20	38.2 ± 1.2	73.4 ± 2.3	0.180 ± 0.010	6017.3	3758.1	498.59	10.38	10.33
2.05	2.21	39.9 ± 1.2	75.6 ± 2.4	0.184 ± 0.011	6098.2	3794.4	504.72	10.51	10.43
3.20	2.22	41.3 ± 1.5	77.2 ± 2.8	0.188 ± 0.013	6163.2	3819.1	508.83	10.60	10.47
7.00	2.23	43.4 ± 1.3	79.5 ± 2.5	0.195 ± 0.011	6272.3	3859.9	514.88	10.72	11.12

To assess structural features on intermediate range, independent analyses were conducted by low-temperature heat-capacity measurements (*C*_p_) and low-frequency Raman spectroscopy. For *C*_p_ analyses, a physical property measurement system (PPMS, Quantum Design) was employed at temperatures down to approximately 2.3 K, using liquid ^4^He. The samples were cut and polished to small discs of about 2 mm in diameter and 1 mm in thickness, corresponding to a mass of about 7 mg. After cleaning, they were fixed on the center of a sapphire platform using ~0.10 mg Apiezon N grease. The estimated error in mass was about ±1 µg, resulting from balance precision. The data of *C*_p_ were measured with the relaxation technique every 10 K within 300 K to 50 K, every 5 K within 50 K to 30 K, every 2 K from 30 K to 20 K, and every 0.5 K from 20 K to about 2 K, applying a logarithmically varying step-size. Within the interval between experimental data points, linear interpolation was conducted as required.

Low-frequency Raman spectroscopy was conducted at room temperature on a confocal micro-Raman spectrometer (Renishaw Invia) equipped with a notch filter for low frequencies (to ~10 cm^−1^). For excitation, a 514.5 nm Argon laser was used. The Raman signal was collected with a CCD camera in the range of 10 to 1375 cm^−1^ with a resolution of 2 cm^−1^, using a 50× objective.

For further reference, infrared reflectance spectra were collected at room temperature on an attenuated total reflection-Fourier transform infrared spectrometer (ATR-FTIR, Perkin Elmer) across the spectral range of 450–7800 cm^−1^ with a resolution of 0.5 cm^−1^, averaged over two individual scans. From these, absorption spectra were derived by Kramers-Kronig transformation, *α*(*v*) = 4*πvk*(*v*), where *α*(*v*) is the absorption coefficient, *k*(*v*) is the imaginary part of the complex refractive index and *v* is the infrared frequency.

Supplementary physical data were collected on density (buoyancy, using ethanol as immersion liquid), elastic constants and sound velocity (ultrasonic echography). For the latter, we used polished discs of ~20 mm in diameter and ~1 mm in thickness. The longitudinal *v*_*L*_ and transversal *v*_*T*_ sound velocities were obtained from the longitudinal and transversal sound wave propagation times, measured with an accuracy of ±1 ns by means of piezoelectric transducers (Echometer 1077, Karl Deutsch, operating at frequencies 8 to 12 MHz). The bulk modulus *K*, Young’s modulus *E* and Poisson’s ratio *ν* were calculated from these data as described elsewhere^18^. All physical data were recorded at room temperature.

## Results

### General

**Figure 1:**
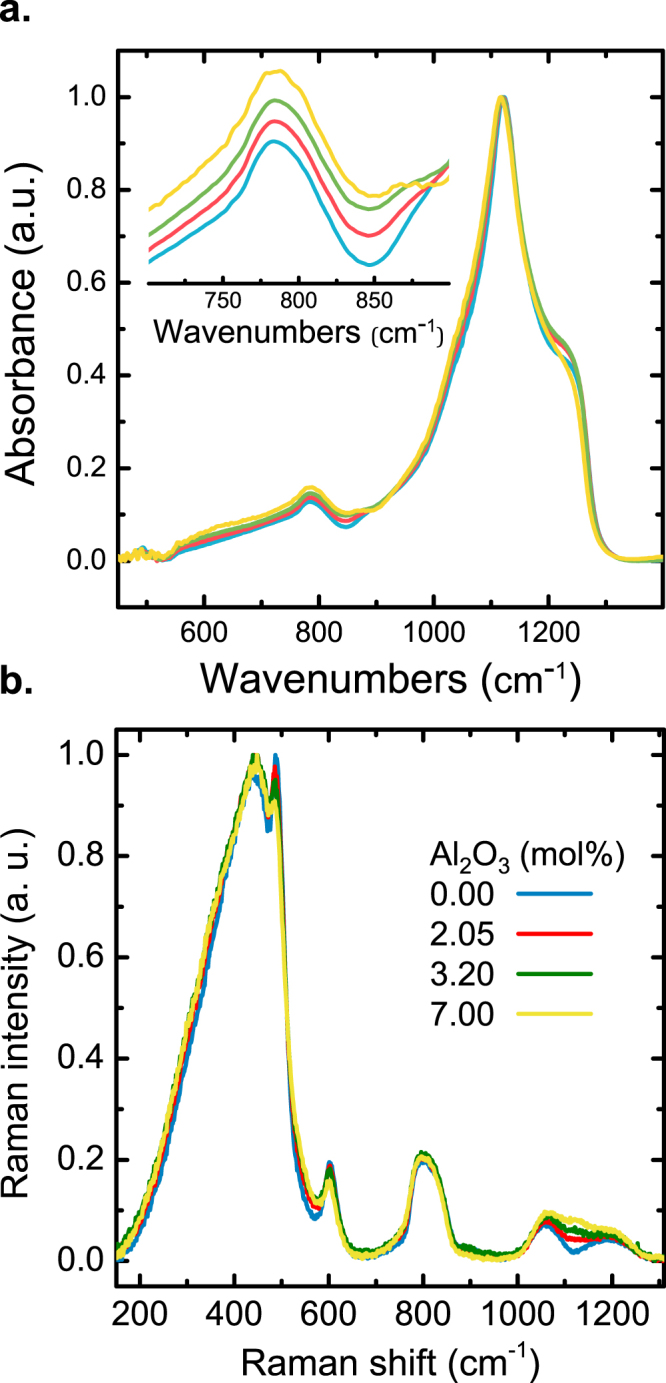
FTIR absorbance (**a**) and Raman scattering spectra (**b**) of silica and binary aluminosilicate glasses. Labels in (**b**) indicate alumina content. The inset in (**a**) is a zoom at the IR absorbance in the spectral region of 700–900 cm^−1^.

IR absorbance spectra of the binary glasses and a silica reference are shown in Fig. 1a. All glasses exhibit similar band shape, dominated by the characteristic vibrations of vitreous silica, *i*.*e*., asymmetric stretching of O-Si-O (~1120 cm^−1^ and 1230 cm^−1^ ^19^) and O-Si-O bending (~800 cm^−1^). The lower band is observed to shift slightly (to lower frequency) when Al_2_O_3_ is present, while the second band broadens somewhat at the same time^20,21^. It was previously suggested that this is related to the extent of disorder in the SiO_2_ network^22^, which seems to increase with the addition of Al_2_O_3_. This interpretation agrees with the gradually disappearing shoulder at 1200 cm^−1^ ^23^.

Corresponding Raman spectra are shown in Fig. 1b. Once again, all glasses exhibit similar band shape, except for the range from 1050 up to 1250 cm^−1^. In the lower frequency region, the band at around 430 cm^−1^ is related to rocking and symmetric bending motions of bridging oxygen (BO) species^24^. The position of this band remains unchanged during the addition of Al_2_O_3_. This indicates that there is no substantial change in the Si-O-Si bond angle.^19^ The sharp peaks at approximately 490 and 600 cm^−1^ correspond to symmetric bridging oxygen bending vibrations in 4- and 3-membered rings^25^. We observe that for both peaks, the relative intensity decreases with increasing Al_2_O_3_ content, further indicating that the addition of Al_2_O_3_ affects the ring-size distribution in the SiO_2_ glass system. In the mid-frequency region, the band at about 800 cm^−1^ results from the motion of Si atoms in their tetrahedral oxygen cage^26^. The high-frequency bands at about 1050–1250 cm^−1^ are usually assigned to Si-O stretching vibrations of tetrahedral SiO_4_ groups. The increasing intensity with addition of Al_2_O_3_ suggests that an interconnected Si-O-Al network is formed.^19^ Also, a new band is arising at around 1100 cm^−1^. It was suggested that this band is associated to Si-O stretching vibrations in SiO_4_ tetrahedra connected to one Al polyhedron.

Since there are no distinct variations in the shape of the characteristic band envelope, we assume here that Al speciation is dominated by four-fold coordination, ^IV^Al. There are several studies that found evidence for the predominance of this coordination state in low-alumina aluminosilicate glasses. For example, Al ions implanted into silica glass (1 × 10^15^–1 × 10^17^ Al^+^ ions cm^2^) were found to simply substitute on Si-positions^27^. Nuclear magnetic resonance (NMR) and extended X-ray absorption fine structure (EXAFS) have shown that for ≤ 0.4 mol% of Al_2_O_3_, the Al ions are predominantly four-fold coordinated^28,29^. Amorphous thin films were investigated by X-ray emission spectroscopy, from which it was concluded that all Al ions are coordinated tetrahedrally for samples containing up to 5 mol % of Al_2_O_3_^30^. Schmücker^31^ has shown by NMR that aluminosilicate glasses containing up to 10 mol% of Al_2_O_3_ contain predominantly four-fold coordinated Al. Finally, Pfleiderer^32^ arrived at the same conclusion through computational simulation of ensembles with up to 13 mol% of Al_2_O_3_.

**Figure 2:**
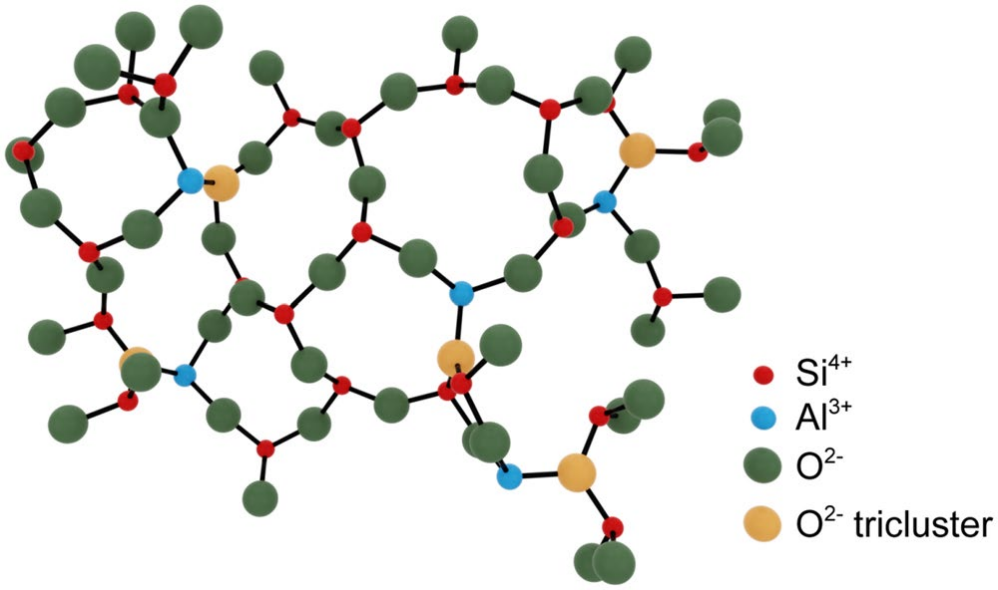
Drawing of the network topology in a binary aluminosilicate glass based on the assumption that all cations are in fourfold coordination and a certain fraction of oxygen triclusters is present. The structure was drawn using the Avogadro software package^42^.

Replacement of one silicon ion for one aluminum ion, without further charge compensation and in tetrahedral coordination, incites one oxygen to bridge three tetrahedra (instead of two) to maintain the charge balance^19,31,33,34^. While the existence of such triclusters is still debated, evidence for their presence in aluminosilicate glasses has been obtained in experiment^35,36^ and through computational simulation^37–39^. In effect, the presence of triclusters in aluminosilicate glasses would induce a tightening of the silica tetrahedral rings and, potentially, initiate crystallization.^34,35^ While triclusters could be bonded either to two Al and one Si or to two Si and one Al^40^, according to the principle of aluminum avoidance proposed by Loewenstein^41^, it is assumed that the latter species is usually dominant. In Fig. 2, an illustration of the structure of binary Al_2_O_3_-SiO_2_ glasses is provided in accordance with these arguments.

### Vibrational Density of States and Boson Peak

The binary of Al_2_O_3_-SiO_2_ exhibits a miscibility gap ranging from ~2–60 mol.% of Al_2_O_3_^43^, giving rise to microscopic heterogeneity of melt-derived glasses^34,44,45^. As for the present case, the presence of phase separation was excluded by high-resolution transmission electron microscopy (TEM) down to a length scale of about 5 nm. Also, on microscopic scale, visible light scattering, Raman microscopic mapping and polarization microscopy did not reveal any heterogeneity. The following observations of low-frequency Raman scattering and low-temperature heat capacity are therefore directly resulting from structural heterogeneity at the nanometric length scale.

#### Specific heat

**Figure 3:**
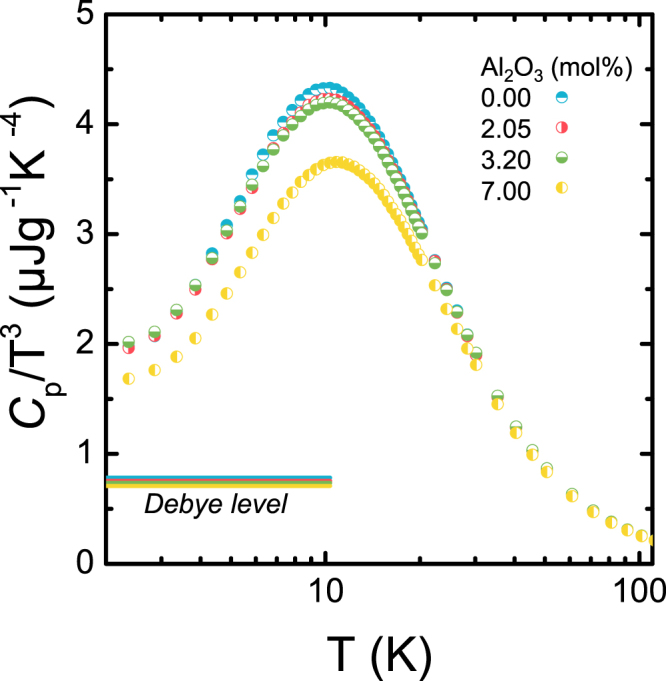
Debye scaling of the temperature dependence of the specific heat of silica and binary aluminosilicate glasses (alumina content indicated in labels). The solid lines in the lower left present the Debye level for each sample according to Eq. 1 (see text for details).

Historically, the attempt to understand non-crystalline environments started from direct comparison with the Debye model of crystals^1^. According to the Debye model, one usually expects a cubic temperature-dependence of the specific heat^46^. However, experimental studies showed a deviation from this behaviour, apparently for the vast majority of non-crystalline materials. This was denoted one of the two *thermal anomalies of glasses*. An early demonstration by Zeller and Pohl^47^ was conducted in vitreous silica. In Fig. 3, a cubic temperature scaling of the heat capacity is displayed for the present samples. The Debye contribution was calculated for each sample according to Eq. (1) and is shown for reference. This uses experimental data of density *ρ*, and transversal *v*_*T*_ and longitudinal *v*_*L*_ sound velocities^48^.

1 $${C}_{Debye}=\displaystyle \frac{2{\pi }^{2}}{5}\left(\displaystyle \frac{{k}_{B}^{4}}{{\hslash }^{3}\rho {v}_{D}^{3}}\right){T}^{3}\approx \frac{234\,{N}_{A}{k}_{B}}{m\,{\theta }_{D}^{3}}{T}^{3}(J/g/K)$$

In Eq. 1, *k*_B_ and $\hslash$ are the Boltzmann and reduced Planck constants, respectively, *N*_*A*_ is Avogadro’s number, *m* is the mean atomic mass in the chemical formula unit, *θ*_*D*_ is the Debye temperature and *v*_*D*_ is the Debye sound velocity obtained by averaging *v*_*L*_ and *v*_*T*_ through $(3/{v}_{D}^{3}).(1/{v}_{L}^{3}+2/{v}_{T}^{3})$. The parameters used for the calculation and the derived values of *θ*_*D*_ are listed in Table 1.

**Figure 4:**
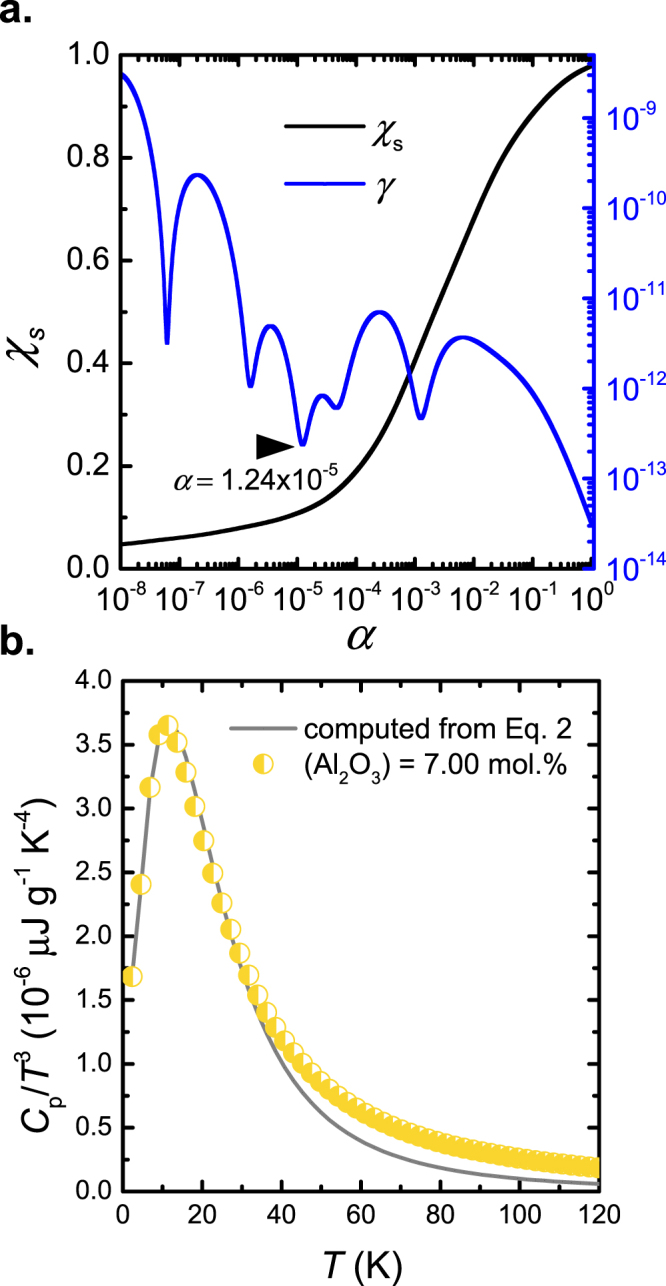
(**a**) Relative residual *χ*_b_ and oscillating parameter *γ* over regularization parameter *α*. For the further fitting, a value of *α* is chosen so that *γ* is minimum while *χ*_*b*_ is acceptably small, indicated in (**a**). (**b**) Corresponding data fit for a sample with (Al_2_O_3_) = 7.00 mol.%, and comparison to experimental data.

All samples exhibit a significant excess of heat capacity over the Debye contribution. This excess takes the form of a broad asymmetric hump, starting at temperatures above 2 K with a maximum intensity at *T*_max_ increasing from about 10.33 to 11.12 K (*T*_max_ values were extracted through a polynomial fit) for increasing alumina content (see Table 1). The shift towards higher temperature is accompanied by a decrease in intensity *I*(*T*_max_). The location of *T*_max_ is directly related to the Boson peak through the connection between the specific heat and the low-energy vibrational density of states (VDoS) *g*(*v*)^48^, with ${C}_{V}={(dU/dT)}_{V}$ leading to^49^

2 $${C}_{p}\approx {C}_{v}=3{N}_{A}{k}_{B}\int g(v){x}^{2}\,\frac{{e}^{x}}{{({e}^{x}-1)}^{2}}dv,$$

where $x=hv/{k}_{B}T$. *C*_*v*_ can be approximated to *C*_*p*_ since glasses are condensed substances in which (*C*_*p*_–*C*_*V*_)*/C*_p_ is less than 0.1%^50^. Eq. 2 presents an ill-posed *inverse mathematical problem* in which the solution is very instable at perturbations. Following Surovtsev *et al*.,^49^ we apply a Tikhonov regularization scheme in order to mathematically stabilize the solution by adding a functional which is weighted with the regularization parameter *α*^49^. However, the choice of *α* is crucial in order to obtain stable solutions without loss of information by excessive smoothing. Knowing that the slope of the reduced density of states *g*(*ω*)*/ω*^2^ in the present VDoS frequency range switches only once from positive to negative (instead of oscillating), we mathematically filter-out the higher frequency solutions. The choice of *α* thus presents a kind of low-pass filtering based on physical knowledge. We used two figures of merit in order to optimize *α*: (i) the relative root-mean-square deviation *χ*_*s*_ and (ii) the integral of the squared derivative of *g*(*v*)/*v*^2^, denoted *γ* and reflecting oscillatory behavior of the solution. Figure 4a shows the variations of these two parameters over *α* for sample containing 7.00 mol.% Al_2_O_3_. Figure 4b shows the corresponding fit for the same sample.

**Figure 5:**
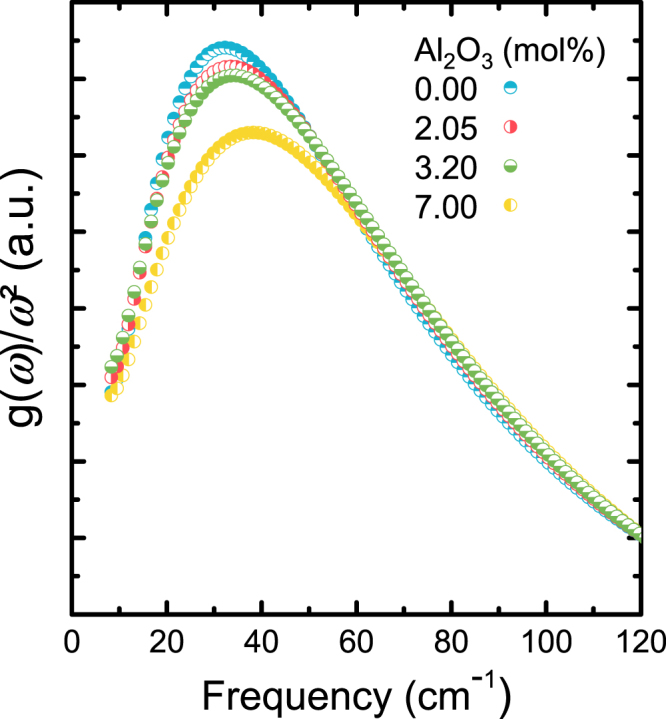
Vibrational density of states normalized over the square of frequency, as obtained by deconvoluting specific heat data of vitreous silica and binary aluminosilicate glasses (alumina content indicated in labels).

The resulting scalings of VDoS are provided in Fig. 5. There is a clear dependence of the excess vibrational density of states on alumina concentration. For the maximum position of the Boson peak of vitreous silica, we find a value of 32.2 cm^−1^ (Heraeus F300). This is close to the value of ~33.5 cm^−1^ as reported in various studies^51,52^ and taken as a confirmation of the accuracy of the present deconvolution across the low-frequency region (<60 cm^−1^).

#### Low-frequency Raman scattering

Prior to the determination of the Boson peak frequency, all Raman spectra were corrected for air scattering and a constant baseline was subtracted individually. In order to compare the intensities of spectra mainly in the low frequency region (*ω* < 175 cm^−1^) the intensity of all samples was normalized to the area under the band peaking at 800 cm^−1^ over the range of 650 cm^−1^< *ω* < 870 cm^−1^ (remaining practically constant with the increasing Al_2_O_3_, within the considered range of compositions). As noted before, this vibrational signature is assigned to the symmetric stretching mode of the O-Si-O bond^53^ or the stretching mode of the Si-O bond.^26^

The measured intensity *I*_*mes*_(ω, *T*) was converted into the reduced Raman intensity through the approach of Shuker and Gammon^54^ (Eq. (3)) to rule-out the temperature-dependence of the Raman spectra. According to the previous model it is possible to connect the reduced density of states *g*(*ω*)/*ω*^2^ to the reduced intensity by means of a coupling coefficient, *C*(*ω*). The reduced intensity of the first order Raman scattering for the Stokes part takes the following form:

3 $${I}^{red}({\rm{\omega }})=\frac{{I}_{mes}({\rm{\omega }},T)}{[{\rm{n}}({\rm{\omega }},T)+1]{\rm{\omega }}}=C({\rm{\omega }})\frac{g({\rm{\omega }})}{{{\rm{\omega }}}^{2}}$$

Here, $n(\omega ,T)={[\exp (\hslash \omega /{k}_{B}T)-1]}^{-1}$ is the Bose-Einstein population factor for frequency *ω* and temperature *T; k*_*B*_ *and* $\hslash$ are the Boltzmann and reduced Planck constants, respectively. The intensity of Raman scattering depends not only on the vibrational density of states, but also on the coupling between photons and vibrations, expressed by the coupling function *C*(*ω*). It is usually calculated by comparison of Raman spectra with neutron inelastic scattering^55^ or through deconvoluting specific heat data.^51^ In the following, we now use the VDoS scaling as described in the previous section for this purpose.

**Figure 6:**
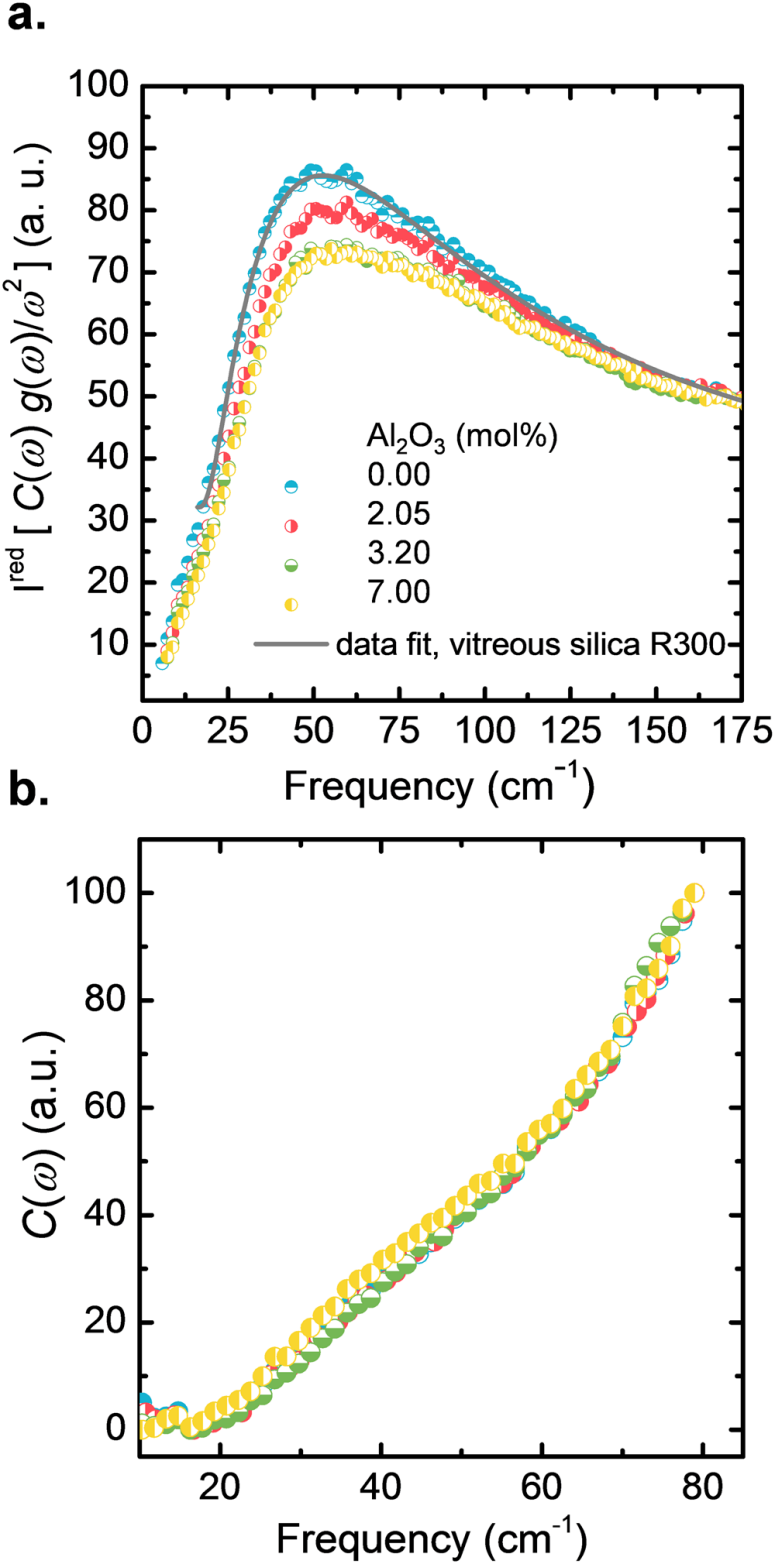
(**a**) Stokes-side of the reduced low-frequency Raman spectra of silica and binary aluminosilicate glasses after subtraction of quasi-elastic scattering (QES) contributions. The solid line represents an exemplary log-normal fit of the experimental data of vitreous silica (type R300) used to estimate the Boson peak frequency. In (**b**), the computed light-vibration coupling coefficient *C*(*ω*) is shown. The alumina content of all samples is given in the labels.

Figure 6a shows the Stokes-side of the reduced low-frequency Raman spectra for various samples of silica and binary aluminosilicate glasses. In the low-frequency region (10–175 cm^−1^) the state density *g*(*ω*) of all the samples exceeds the prediction of the Debye model for an isotropic homogeneous continuous medium. This excess of modes appears as a broad peak between 53–60 cm^−1^ when *g*(*ω*)/*ω*^2^ is plotted over frequency (with the Debye density of states ${g}_{{\rm{Deb}}}(\omega )\approx {\omega }^{2}$. The maximum of this peak, ${\omega }_{BP}$, shifts toward higher frequency and its intensity decreases with increasing Al_2_O_3_ content. To obtain quantitatively the Boson peak frequency, it is necessary to use a function which reproduces-well the reduced intensity *I*^*red*^ (ω) and takes into account the asymmetric shape of the Boson peak. For this, we used a log-normal function^56^,

4 $$I({\rm{\omega }})={\rm{H}}\,\exp (-\,\mathrm{ln}(2){[\frac{\mathrm{ln}(1+2a\frac{{\rm{\omega }}-{{\rm{\omega }}}_{BP}}{w})}{a}]}^{2})+{I}_{0}$$

In Eq. 4, *H* is the amplitude, *a* is the asymmetry parameter, *ω* is the width of the log-normal function and *I*_0_ is an off-set parameter.

In Fig. 6b the light-vibration coupling coefficient is shown. It varies almost linearly with *ω* for all samples, similar the earlier observations on other glasses^51,57^. For the moment, we hence assume that there is no significant effect of *C*(*ω*) on the interpretation of the Boson peak in the glass types of this study. Furthermore, we note the uncertainty in *C*(*ω*) on the high-frequency tail of the Boson peak which increases with increasing frequency (*ω* > 60 cm^−1^). This uncertainty is attributed to the inaccuracy of the computed *C*_p_ data according to Eq. 2 as shown in Fig. 4b for T > 40 K.

**Figure 7:**
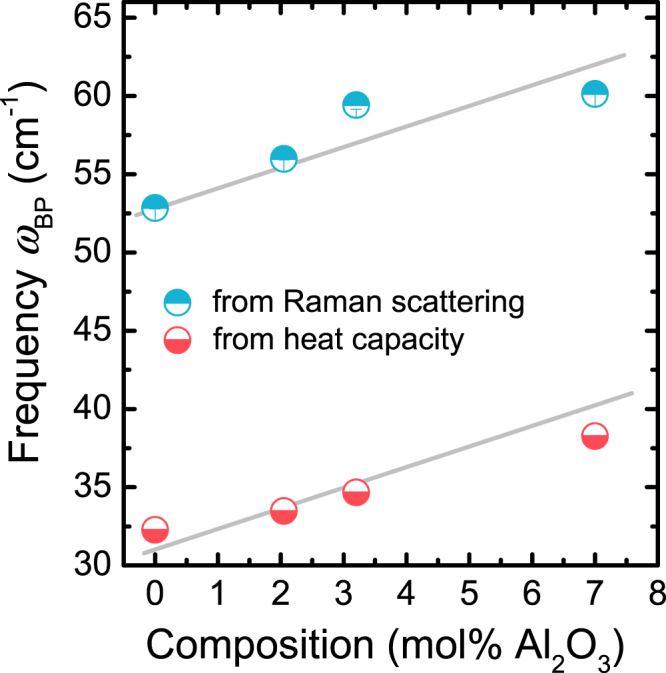
Variation of the Boson peak frequency ω_*BP*_ extracted from Raman low-frequency scattering and deconvolution of heat capacity, respectively, as a function of Al_2_O_3_ content in binary aluminosilicate glasses. Lines are drawn to guide the eye.

The evolution of the Boson peak frequency ω_*BP*_ as extracted from low-frequency Raman scattering and deconvolution of heat capacity data, respectively, can now be explored as a function of Al_2_O_3_ content (Fig. 7). We compared the variation of the Boson peak frequency ω_*BP*_, extracted from Raman scattering at low frequencies and through deconvolution of heat capacity, as a function of Al_2_O_3_ content. In both cases, a clear trend is found where ω_*BP*_ increases with the addition of Al_2_O_3_. Also, for all samples, the values of ω_*BP*_ extracted from Raman low-frequency data are greater than those extracted from the deconvolution of heat capacity data. However, the absolute shift of the Boson peak frequency ${{\rm{\Delta }}{\rm{\omega }}}_{BP}={{\rm{\omega }}}_{BP}(93\,\,{{\rm{SiO}}}_{2}-7\,{{\rm{Al}}}_{2}{{\rm{O}}}_{3})-{{\rm{\omega }}}_{BP}({{\rm{SiO}}}_{2})$ in both datasets remains unchanged, *i*.*e*., ${{\rm{\Delta }}{\rm{\omega }}}_{BP}\approx 6\,c{m}^{-1}$.

## Discussion

### Vibrational density of states and Boson peak

The excess of heat capacity *C*_p_(*T*)/*T*^3^ relative to the level predicted by the Debye model of thermal properties for silica and aluminosilicate glasses is shown in Fig. 3. Not visible in the present data, due to the limited temperature regime, there are indeed two temperature intervals in which such excess occurs: First, at very low temperatures (<1 K), *C*_p_ varies linearly with *T* (*C*_p_ ∝ a*T*, contrary to the crystallized form of silica in which *C*_p_ ∝ a*T*^3^)^47^. This anomaly is commonly explained on the basis of a two-level system (TLS) in which disorder results in structural defects. Tunneling from one configuration to another then causes additional contributions to *C*_p_^58,59^. As for the present study, the TLS regime is not explored because we did not consider temperatures lower than 2 K. As noted before, we treat the second regime of anomaly in which the Boson peak is found instead.

Both anomalies were successfully explained by the soft potential model (SPM) developed by Karpov and Parshin^60,61^ and by Yu and Freeman^62^ over the temperature range of 0 <T <(3T_min_ + T_max_)/4 ^63^ (where *T*_min_ and *T*_max_ are the temperature of the minimum and maximum in *C*_p_(*T*)/*T*^3^). This model assumes that the low-frequency dynamics of glasses are characterized by the presence of additional quasi-local harmonic oscillators (soft modes) that coexist and interact with ordinary (delocalized) phonons. The further postulates that the total specific heat can be written as *C*_p_ = *C*_TLS_*T* + *C*_D_ *T*^3^ + *C*_sm_ *T*^5^ ^64^, where *C*_TLS_, *C*_D_ and *C*_sm_ represent the contributions of TLS modes, Debye modes and soft mode vibrations, respectively. For an indication, values for amorphous silica were reported at *C*_TLS_ = 3.15 μJ/gK^2^, *C*_D_ = 0.76 μJ/gK^4^ and *C*_SPM_ = 0.11 μJ/gK^6^ ^63^ in the range of 0–4.15 K (with *T*_min_= 2.1 K^65^).

In Figs 5 and 6a, the density of states *g*(*ω*) for all samples exceeds the prediction of the Debye model. This excess of modes is often also visible in inelastic neutron scattering (INS), X-ray scattering (IXS), hyper-Raman scattering, infrared spectroscopy (IR) and terahertz time-domain spectroscopy (THz-TDS). Its microscopic origin is not well understood. For the specific case of vitreous silica, Buchenau^66^ argued on the basis of elastic and inelastic neutron scattering that the excess of modes giving rise to the Boson peak is produced by rotational motions of several interconnected tetrahedral SiO_4_ units. This phenomenon was also demonstrated by Hehlen *et al*.^67^ using hyper-Raman spectroscopy. Based on this, Richet^68^ concluded that the origin of the Boson peak in binary silicate glasses could be the result of the sum of two independent contributions, *i*.*e*., rotational motions (liberation modes) of interconnected tetrahedral SiO_4_ units and localized vibrations of the network modifier cations within oxygen cages. Noteworthy, the latter are different from the common cage rattling modes of modifier cations as they are typically found in the Raman scattering range of 100–300 cm^−1^ ^69,70^.

Besides the experimental deductions, various theoretical approaches exist to explain the microscopic origin of the excess of modes. For example, the model of the non-continuous structure of Duval *et al*.^71^ suggests that amorphous materials comprise of nanometric domains (heterogeneities) in addition to the fundamental building blocks (*e*.*g*., SiO_4_ tetrahedra). The links between two neighboring atoms would be stronger if they belong to the same domain (cohesive domains) as compared to neighbors in distinct domains (soft domains). According to this model, the Boson peak is a signature of hybridization of acoustic and localized modes^72^.

### Heterogeneity and intermediate-range order

Figure 3 shows a negative correlation between excess intensity (*C*_p_/*T*^3^)_max_ and *T*_max_. This observation is in agreement with the study of Liu *et al*.^73^ who explored various amorphous materials: inorganic oxide, metallic and organic polymer glasses. According to their results the noted negative correlation is a general feature for amorphous systems. The value of (C_p_/*T*^3^)_max_ is strongly influenced by the nature and size of the network-modifying cation^55,68,74,75^. For example, Nakamura^75^ classified the respective cations according to their ionic radii. However, it was also noted that the ionic radius of the cation cannot be the only factor which is governing the Boson peak intensity because cations with similar ionic radius may exhibit different behavior.^69^

A clear trend of increasing Boson peak frequency ω_*BP*_ and decreasing intensity (*g*(*ω*)/*ω*^2^)_*max*_) (Fig. 5) and ${({I}^{red})}_{max}$ (Fig. 6a) with increasing Al_2_O_3_ content is observed. Similar variations have previously been related to increasing stiffness induced by several parameters, including density^76^, pressure^77^ or network connectivity^78^. The intensity decrease with the increase of Al_2_O_3_ content could be explained by the fact that the contribution of the localized vibrations of the Al cation relative to non-bridging oxygen species is less important than the contribution of liberation of interconnected tetrahedral SiO_4_.^74^ Indeed, when more Al_2_O_3_ is introduced into the network the sum of the two contributions becomes less pronounced.

Nakamura *et al*.^79^ were interested in the effect of introducing network modifying cations on the nature of the Boson peak, conducting their study on binary alkali and alkaline-earth silicate glasses of the type (100-x) SiO_2_−xM [M = Li_2_O, CaO, Na_2_O, K_2_O, BaO]. They found two trends in which the size of the ions introduced into the network either increases (*r*_ion_/*r*_Si_<2.5) or decreases (*r*_ion_/*r*_Si_>2.5) the Boson peak. This was similar to an earlier study^80^ in binary alkali silicate glasses (100-x) SiO_2_−xM [M = Rb_2_O, Cs_2_O]. Nakamura *et al*. also found^79^ a linear relationship between the Boson peak frequency ω_*BP*_ and the mean atomic volume V_M_/Å^3^ representative for the interatomic distance: ω_*BP*_ decreases when V_M_/Å^3^ increases. In the present data, we confirm this trend. We have *r*_Al_/*r*_Si_ = 1.25 and V_M_/Å^3^ = [15.10, 15.01, 14.98, 14.93] for samples containing [0, 2.05, 3.20, 7.00] Al_2_O_3_ mol.%, respectively. For ternary sodium aluminosilicate glasses, it was further noted that Al_2_O_3_ replacing Na_2_O increases the Boson peak frequency due to distortion of the SiO_4_ tetrahedral network^81^.

**Figure 8:**
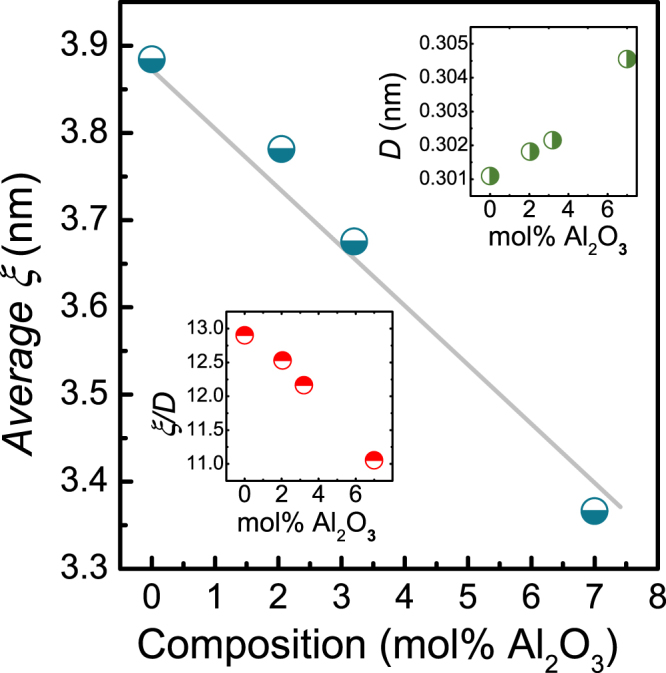
Variation of the dynamic correlation length *ξ* in silica and binary aluminosilicate glasses as a function of alumina content. The mean inter-particle distance *D* is shown in the upper inset, and the ratio *ξ/D* in the lower inset. The line is drawn to guide the eye.

Building on these previous observations, the present trend is explained on the basis of the elastic proprieties of binary aluminosilicate glasses. Al_2_O_3_ acts on the local symmetry of tetrahedral SiO_4_ stretching motions^82^, decreasing the degrees of freedom in the system and increasing stiffness. This reflects in increasing bulk modulus (Table 1). Further understanding is derived from the inhomogeneity models explained above. Duval^71^ and Elliott^83^ independently found a relationship that links the average size of heterogeneities ξ with the position of the Boson peak frequency ω_*BP*_ through the relation

5 $${\rm{\xi }}={v}_{T}/{{\rm{\omega }}}_{BP},$$

where *v*_*T*_ is the transverse sound velocity. Figure 8 displays the variation of the average length scale of heterogeneity extracted from the deconvolution of heat capacity data as a function of Al_2_O_3_ content. We find a decrease of the average size of heterogeneities *ξ* from 3.88 nm in *v*-SiO_2_ (compared to 3.74 nm as reported previously^63^) to 3.36 nm for an alumina content of 7 mol.%. To understand this behavior, we compare *ξ* to the mean inter-particle length *D* $(D={[M(x)/({N}_{A}\times \rho (x))]}^{1/3})$. The results shown in the insets of Fig. 8 indicate that the value of $D$ increases with increasing content of Al_2_O_3_, probably associated with the presence of triclusters. The ratio of ${\rm{\xi }}$/D decreases with increasing Al_2_O_3_ content, which is a signature of the increase of elastic homogenization at the nanometric scale and translates into decreasing contrast between strong and soft domains.

### Boson peak shape and scaling

**Figure 9:**
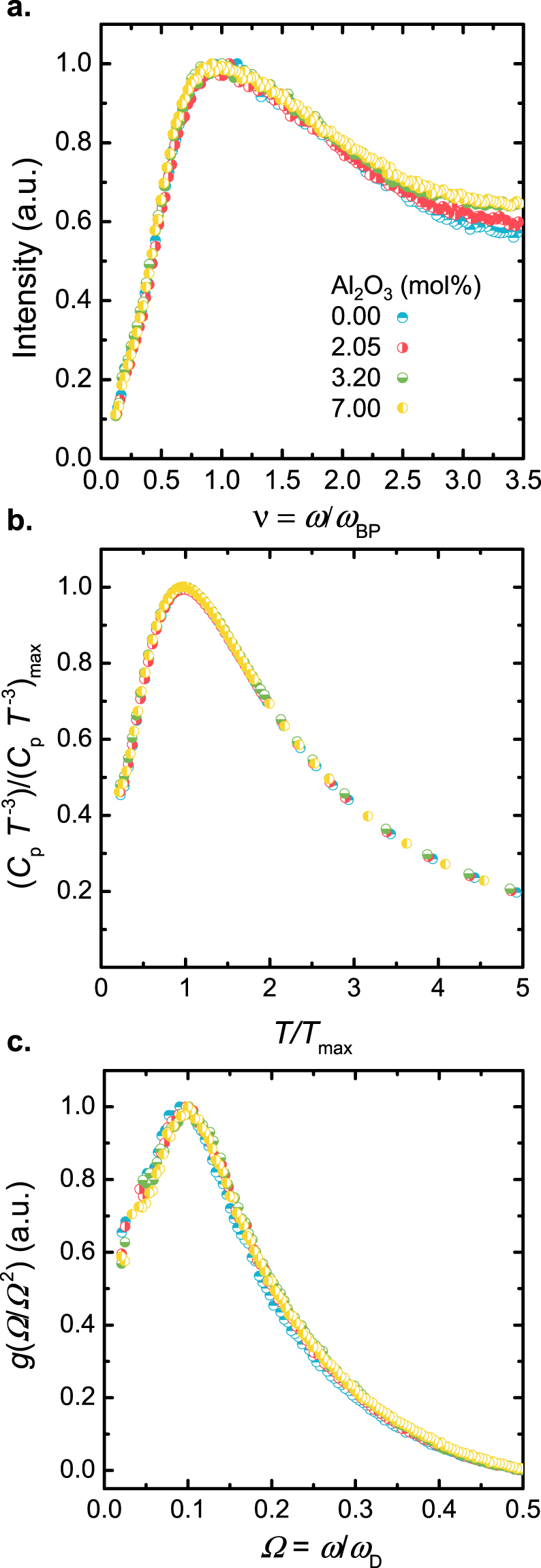
Normalized master curve of the Boson peak for frequency-scaling of the Raman scattering intensity (**a**) and temperature-scaling of the isobaric heat capacity (**b**). (**c**) Shows the set of curves which are obtained through scaling of the Boson peak with the reduced density of vibrational states after normalizing with the Debye cut-off frequency ${{\rm{\omega }}}_{D}$. Value of ${{\rm{\omega }}}_{D}$ a function of Al_2_O_3_ content are provided in Table 1.

In order to further elaborate on the hypothesis that the decrease of Boson peak intensity and its simultaneous shift to higher frequency (and higher *T*_max_) are resulting from changes of elastic properties, we consider the shape of the Boson peak as a function of composition. As already noted, it has been found in many studies that changes in chemical composition, chemical bond type (covalent or metallic), density, pressure and temperature lead to changes in the Boson peak. When plotted in reduced form, these changes are universal and can be summarized in a single master curve^84^. We now carry-out different scaling approaches. First, we consider the Boson peak as function of the scaled frequency $\upsilon$, obtained by reducing the frequency axis, ${\rm{\upsilon }}={\rm{\omega }}/{{\rm{\omega }}}_{BP}$, and normalization of the VDoS using $g(\upsilon ){\rm{d}}\upsilon =g(\omega ){\rm{d}}\omega$ (Fig. 9a). This change of variable from $\omega$ to $\upsilon$ must be accompanied by a scaling of the reduced Raman intensity. Assuming $C(\omega ) \sim \omega$, the rescaled intensity can be written as

6 $$I(\nu )={I}^{red}(\omega )\times {\omega }_{BP}^{2}$$

In a second approach, the results of the heat capacity were also scaled (Fig. 9b). Here, the scaling was performed by dividing the intensity by the maximum value (*C*_p_ *T*^−3^) _max_ and *T* by *T*_max_. For both approaches, the scaled spectra (corresponding to different glass compositions) fall on a same master curve without introducing any adjustment parameter.

We now compare the evolution of the VDoS to the elastic medium transformations. The latter are quantified by calculating the Debye frequency ${{\rm{\omega }}}_{D}=(6{\pi }^{2}\rho {N}_{A}m/M){}^{1/3}{v}_{D}$ (*M* is the average molar weight). The excess of vibrational density $g(\omega )/{\omega }^{2}$ is extracted by subtracting the contribution of the coupling factor already calculated from the reduced intensity ${I}^{red}$ (Eq. 3), and again scaling with the Debye frequency ${{\rm{\omega }}}_{D}$. Therefore, the frequency axis of the excess of VDoS spectra obtained from the conversion of heat capacity data (reported above) was rescaled as *Ω* = *ω*/*ω*_*D*_. To conserve the total number of vibrational states^85^, the intensity of the spectra must be also rescaled as

7 $$\frac{g(\Omega )}{{\Omega }^{2}}=\frac{g({\rm{\omega }})}{{{\rm{\omega }}}^{2}}{{\rm{\omega }}}_{D}^{3}$$

These rescaled spectra are displayed in Fig. 9c.

In all three scalings, the shape of Boson peak remains practically unchanged across all samples. This agrees with earlier observations on various other glasses. We thus conclude that the vibrational modes responsible for the Boson peak are the same in silica and aluminosilicate glasses. The universal shape observed in the Fig. 9c. can be related to the universality of the spatial distribution of fluctuations in the elastic constants^86^. Furthermore, the scalability is solely determined by changes occurring in the elastic properties of the material. On the other hand, failure in rescaling of the Boson peak to a single master curve has been attributed microscopic modifications^87^ such the existence of several phases in the same material (phase separation)^88^.

## Conclusions

In summary, we considered the low-frequency vibrational modes of binary aluminosilicate glasses relative to a reference of vitreous silica. In such materials, heterogeneity on intermediate length scale is a crucial factor for optical fiber performance, determining the lower limit of optical attenuation and Rayleigh scattering, but also clustering and precipitation of optically active dopants, for example, in the fabrication of high-power laser gain media. Using low-temperature heat capacity and low-frequency Raman scattering data, we obtained an accurate scaling of the vibrational density of states and the Boson peak. This allowed for the extraction of the average dynamic correlation length as a function of alumina content. In the absence of macroscopic phase separation, it was found that this value decreases with increasing alumina content. At the same time, the average inter-particle distance increased slightly, assumedly associated with the presence of oxygen tricluster species. In accordance with Loewensteinian dynamics, mild alumina doping therefore increases structural homogeneity on molecular scale.

### Data Availability Statement

The datasets generated during and/or analysed during the current study are available from the corresponding author on reasonable request.
